# The effect of neuropalliative care on quality of life and satisfaction with quality of care in patients with progressive neurological disease and their family caregivers: an interventional control study

**DOI:** 10.1186/s12904-020-00651-9

**Published:** 2020-09-16

**Authors:** Radka Bužgová, Radka Kozáková, Michal Bar

**Affiliations:** 1 Department of Nursing and Midwifery, Faculty of Medicine, University of Ostrava, Syllabova 19, 700 30 Ostrava, Czech Republic; 2 Neurology clinic, The University Hospital Ostrava, Ostrava, Czech Republic

**Keywords:** Quality of life, Palliative care, Symptoms burden, Subjective assessment

## Abstract

**Background:**

It is recommended that patients with progressive neurological disease (PND) receive general and specialized palliative care. The purpose of this study was to determine the effect of neuropalliative care on quality of life (QoL) and satisfaction with provided care in both patients with PND in advanced stages of disease and their family caregivers.

**Methods:**

The sample consisted of 151 patients with PND and 140 family caregivers. The PNDQoL questionnaire was used for data collection. Patients and family caregivers completed the questionnaires both before and 3 months after the intervention.

**Results:**

Before intervention, there were no statistically significant differences in the individual domains of QoL in patients and family caregivers in either the intervention or the control group. After intervention, differences were identified in the sample of patients in the domains of **symptoms burden** (*p* < 0.001), **emotional** (p < 0 .001), **social functioning** (*p* = 0.046), **spiritual area (nonreligious)** (*p* = 0.050), and in **QoL**. In the sample of family caregivers, there were differences in the domains of **symptoms burden** (*p* < 0.001), **emotional functioning** (*p* = 0.016), **spiritual area (nonreligious)** (*p* = 0.042), and in the **assessment of health** (*p* = 0.002), and **QoL** (p = 0.002). Patients and family caregivers from the intervention group evaluated their satisfaction with the quality of care provided significantly more positively in all five analyzed domains.

**Conclusion:**

The provision of neuropalliative care to patients with advanced stages of PND helped to maintain and slightly improve their QoL, and symptoms burden, and resulted in a more positive assessment of satisfaction with the quality of care provided.

## Background

Despite developments in scientific knowledge in the fields of neurology and molecular biology, and significant progress in the treatment of patients with progressive neurological disease (PND), there are still many treatment procedures, especially for patients with motor neuron disease (MND), Parkinson’s disease (PD), multiple sclerosis (MS), or other neurodegenerative diseases, that are palliative, rather than curative. Such treatment focuses on the reduction of symptoms, improving quality of life, and, in some cases, prolongation of the patient’s life [1]. The concept of neuropalliative and rehabilitative care suitable for patients with PND is defined by Sutton [2] as “a holistic approach to the care of neurological patients with significant disability, complex needs, and a potentially shortened life-span. It is patient-centered and involves diagnosis of clinical problems at all stages, rehabilitation to maintain function, care coordination and appropriate palliation to relieve symptoms”. Recently, an increasing number of authors have focused on the integration of palliative care into neurology [3–6].

The European Federation of Neurological Societies (EFNS) in its key neurological guidelines also recommends that all patients with PND are provided with multidisciplinary care, and have access to both general and specialized palliative care [7].

For neurological patients, the most appropriate forms appear to be what are known as “early” palliative care and the “dynamic model” of palliative care [8, 9]. Early palliative care does not exclude the concurrence of curative and palliative care, the intensity of palliative care services gradually increasing as the disease advances. In the dynamic model, specialized palliative care services are provided based on trigger points, i.e., at intermittent periods according to the patients’ needs [8, 10]. The aim of palliative care in all stages of disease should be to achieve the optimum quality of life for patients and their families [11, 12]. On the other hand, specialized palliative care should be provided in the form of complex interventions which positively impact the QoL of patients and their family members, reduce the burden of physical symptoms, and have a positive effect on psychosocial and spiritual issues, individual preference of care, and the quality of death and dying [9]. Analysis of patients’ subjective perception of the impact of disease and treatment on activities of daily living, ability to self-care, emotional experiences, social relationships, and level of anxiety, tension, and depression is very important for the evaluation of the quality of care provided to patients with PND.

Based on a systematic review and meta-analysis, Kavalieratos et al. [13] found that palliative care intervention (e.g. symptom management, communication and goal setting, carer support, end of life care) is associated with improved quality of life of patients with life-limiting disease, and a mitigated symptoms burden. However, they did not find any connection between palliative care and survival. Few studies have been published analyzing the positive effects of neuropalliative care on the quality of life of patients with PND and their family caregivers, or on satisfaction with provided care. Several studies have indicated improved quality of life, and reduced pain, dyspnea, sleep disorders, and bowel symptoms, after the application of palliative care to patients with amyotrophic lateral sclerosis [14, 15], multiple sclerosis [16], Parkinson’s disease [17], neurodegenerative diseases [18]; and, in addition, a reduced burden for family caregivers [16]. A randomized study confirmed the financial efficiency of early palliative care in patients with multiple sclerosis [19]. Oliver and Veronese [9] recommend further research to focus on the effect of complex specialized palliative care interventions on all aspects of quality of life of patients with PND.

There is also a lack of studies confirming the satisfaction of neurological patients and their family caregivers with the palliative care interventions provided. In terms of palliative care, only the satisfaction of oncological patients has been assessed in a study [20–25].

The aim or our research was to analyze the effect of neuropalliative care of patients with PND in advanced stage of disease (PPS - Palliative Performance Scale ≤70 points) and their family caregivers on their quality of life and their satisfaction with the care provided. Another aim was to determine predictors of change in quality of life of patients and their family caregivers.

## Methods

### Design

We performed an interventional study – randomized controlled trial study design. Interventional study is specifically tailored to evaluate direct impacts of treatment measures on disease. Randomized controlled trials are the most common type of interventional study [26].

### Participants

The sample included 291 participants (151 patients with PND and 140 family caregivers) who met the following criteria:

- Sample of patients with PND: diagnosed with selected PNDs according to ICD-10: multiple sclerosis (G-35), Parkinson’s disease (G-20), motor neuron disease (G-12.2); aged 18+ years; PPS (Palliative Performance Scale) ≤ 70 points; MMSE (Mini-Mental State Examination) ≥ 24 points; and consenting to participation by signed informed consent.
- Sample of family caregivers: primary family caregivers; aged 18+ years; and consenting to participation by signed informed consent.

Patients with PND and their family caregivers were recruited via four institutions in the Moravian-Silesian region of the Czech Republic: 1. the Neurology Department of University Hospital, Ostrava (the Therapeutic Center for Demyelination Diseases and the outpatient department for extrapyramidal diseases and cognitive disorders); 2. Chuchelná Rehabilitation Institute; 3. social institutional care (residential care home for the elderly); and 4. a nursing home in Ostrava. A doctor or head nurse from each facility selected all suitable patients with PND, based on the stated criteria, who were then invited to join the study. Having consented to participation, the patients with PND were then placed randomly either in the intervention group or in the control group, according to the final digit in their social security number – National ID number (odd/even). Categorization was performed by a doctor or the study coordinator. Afterwards, family caregivers were also invited to participate, and were placed in the same group as their patients (intervention/control group). Eleven family caregivers chose not to participate in the project. They don’t agree with participation because they didn’t want to fill the questionnaires.

### Intervention

The research involved an interventional control study. The intervention consisted of neuropalliative care in the form of multidisciplinary palliative team consultations. A four doctors (neurologist) evaluated the functional scales (EDSS – Expanded Disability Status scale [27] for multiple sclerosis patients; ALS functioning rating scale for patients with amyotrophic lateral sclerosis [28]; Schwab and England Activities of Daily Living scale for patients with Parkinson’s disease [29]; while the PPS (Palliative Performance Scale) [30] and ADL (Activities of Daily Living) [31] were evaluated for all patients. The doctors were not part of the multidisciplinary care team.

Patients with PND and family caregivers were asked to complete a quality of life evaluation questionnaire (Time I). Then the study coordinator contacted the patients from the intervention group to offer them a consultation with a multidisciplinary palliative team (doctor – palliative care physician, nurse, social workers, psychologist, priest, physiotherapist, speech therapist, and occupational therapist). Based on the patient’s needs, the intervention was provided in the form of individual consultations with a specific expert, in which family caregivers could also participate. The duration of a consultation was 45–60 min. The consultations were provided in the outpatients’ department, in the facility or the home of the client. The doctor’s consultations were focused on mitigating the symptoms (pain, other symptoms causing suffering) and dealing with the patient’s discomfort including medication adjustments, formulating a plan of care, and objectives of treatment during the individual stages of the disease.

The physiotherapist and the occupational therapist discussed the functional objectives (self-care, limits to self-sufficiency), respiratory and cognitive rehabilitation, care focusing on spasticity and mobility, timely provision of proper equipment. The speech therapist recommended care focusing on the option of alternative communication in case of speech difficulties. The psychologist offered help with the adaptation to the disease, managing one’s emotions, social changes, and the increased dependency on the help of others, including the support of maintaining one’s dignity. Furthermore, the psychologist focused on the connection between the physical symptoms and the mental state of the patient. The social worker discussed the options of financial support and securing the social services. The team members were not from some health care facilities. The team coordinated the study coordinator, and the professional communicated with each other by phone. The average number of professionals who consulted with one patient and their family caregivers was 3.4.

The control group patients were provided with standard care. Standard care included routine care by a neurologist (routine visit in an outpatient setting or institutional care). The neurologist consulted symptoms and prescribed medication. The nursing assistant care was provided to patients who lived in a nursing home or social institutional care. In these facilities, the standard care was provided by a neurologist who visited the facility externally.

After 3 months, the patients with PND and family caregivers were asked again to complete the quality of life evaluation questionnaire and a questionnaire analyzing their satisfaction with provided care (Time II). A paper-and-pencil version of the questionnaires could be completed in the facilities or taken home and returned in person or by mail.

The patients in the control group could consult with a multidisciplinary palliative team after the study.

### Data collection

**Quality of life** was measured with the PNDQoL [32] questionnaire (Progressive Neurological Diseases Quality of Life), with versions for patients and family caregivers. It was developed and validated in our research project. We created a questionnaire specifically for patients with PND and their caregivers in Czech version, which would reflect the specific problems of these patients and their family caregivers in the context of the Czech environment. Appropriate psychometric characteristics were verified. Acceptable reliability was found in all domains of the questionnaire for patients with PND (α = 0.727–0.834) and family caregivers (α =0.735–0.923), [32].

The PNDQoL includes symptomatic and functional scales, and assessment of global health and global QoL.

The symptomatic scale for patients asks them to rate 11 symptoms (pain, fatigue, sleepiness, dyspnea, tremor, stiffness, spasms, swallowing, salivation, excretion, and urination) from 0 (none) to 10 (severe); while the version for family caregivers asks them to rate ten physical symptoms (pain, tiredness, sleep dysfunctions, pounding heart, dyspnea, problems with food intake, nausea, skin problems, sexual problems, and excretory problems) from 0 (none) to 4 (very many). The overall scores for the two symptomatic scales can range from 0 to 110 points (patients) and from 0 to 40 points (family caregivers). The higher the number of points, the greater the burden of symptoms.

The functional scale of QoL (32 items) is divided into four domains (1. activities of daily living, 2. emotional functioning, 3. social functioning, and 4. spiritual areas), with each domain containing eight items. The items are evaluated on a 5-point Likert scale ranging from 0 (not at all) to 4 (very often). The score for the domains ranges from 0 to 100. A higher number represents a greater burden, and, therefore, worse QoL in the given area. Evaluation of the fourth domain, “spiritual areas”, is divided into two parts: 4a “nonreligious”, and 4b “religious”.

The assessment of global health and global QoL is made through a single item with a 10-point scale ranging from 1 (very bad) to 10 (excellent).

**The severity of the disease** was measured with the following scales: the EDSS – Expanded Disability Status scale [27], the PPS – Palliative Performance Scale [30], and the ADL – Activities of Daily Living [31]. In terms of sociodemographic information, we traced age, gender, marital status, education, and children – yes/no.

**Evaluation of satisfaction with provided care** was performed with a modified version of the CANHELP Lite questionnaire [33], which includes 21 items grouped in five domains: 1. **Global satisfaction** (1 item), 2. **Relationship with doctors** (2 items), 3. **Satisfaction with the care of health workers** (4 items), 4. **Disease management** (7 items), and 5. **Communication and decision making** (7 items). The items are evaluated on a 5-point Likert scale ranging from 1 (totally dissatisfied) to 5 (totally satisfied). Domain scores range from 20 to 100. A higher score represents greater satisfaction with care in the domain area. Using Cronbach’s alpha coefficient (α), the reliability of the Czech version of the questionnaire was evaluated as satisfactory (patients’ domains: α = 0.830–0.914; family caregivers’ domains: α = 0.863–0.939).

### Data analysis

The normality of data distribution was tested by means of the Shapiro-Wilk test. The data are depicted through descriptive statistics – mean, standard deviation, and relative and absolute frequency. To evaluate the differences between the groups, the non-parametric Wilcoxon two-sample test for independent samples (Mann-Whitney U test), and Wilcoxon singed-rank test were used. The predictors influencing changes in QoL were tested through multiple linear regression analysis. The following are the predictor variables for patients: palliative intervention, gender, age, marital status, and PPS score; and for family caregivers: intervention, gender, age, marital status, and patients’ PPS score. The statistical significance was tested with the significance level *p* < 0.05. The data were analyzed using the software SPSS v. 24.0 (IBM, Armonk, NY, USA).

## Results

### Sociodemographic characteristics of the sample

A total of 151 patients with PND (98 in the intervention group, and 53 in the control group) and 140 family caregivers (93 in the intervention group, and 47 in the control group) participated in the study. The family caregivers were most often the life partners of the patients (intervention: 43%, control: 55.3%), their children (intervention: 36.6%, control: 29.8%), parents (intervention: 11.8%, control: 12.8%), or other relatives (intervention: 8.6%, control 2.1%). The sociographic characteristics of the sample are depicted in Table 1.

**Table 1:** Sociodemographic characteristics of the sample

	Patients with PND			Family caregivers		
	interventional *N* = 98	control *N* = 53	total *N* = 151	interventional *N* = 93	control *N* = 47	total *N* = 140
**Age** N (%)						
mean (SD)	62.6 (13.4)	66.3 (11.5)	63.9 (12.8)	52.7 (14.9)	58.1 (14.4)	54.5 (14.9)
Min–max	38–90	43–91	38–91	22–84	26–85	22–85
**Gender** N (%)						
Man	39 (39.8)	22 (41.5)	61 (40.4)	38 (41.3)	17 (36.2)	55 (39.6)
Women	59 (60.2)	31 (58.8)	90 (59.6)	55 (58.7)	30 (63.8)	85 (60.4)
**Marital status** N (%)						
Single	11 (11.2)	3 (5.7)	14 (9.3)	18 (19.6)	6 (12.8)	24 (17.3)
Married	48 (49.0)	31 (58.5)	79 (52.3)	67 (71.7)	33 (70.2)	100 (71.2)
Divorced	20 (20.4)	6 (11.3)	26 (17.2)	6 (6.5)	7 (14.9)	13 (9.4)
Widow/er	19 (19.4)	13 (24.5)	32 (21.2)	2 (2.2)	1 (2.1)	3 (2.2)
**Education** N (%)						
Elementary	17 (17.3)	11 (20.8)	28 (18.5)	4 (4.3)	10 (21.3)	14 (10.0)
Secondary	69 (70.4)	38 (71.6)	107 (70.8)	62 (66.6)	28 (59.6)	90 (64.3)
Higher	3 (3.1)	0 (0.0)	3 (2.0)	15 (16.1)	5 (10.6)	20 (14.3)
Tertiary	9 (9.2)	4 (7.5)	13 (8.6)	12 (12.9)	4 (8.5)	16 (11.4)
**Employment N** (%)						
Employee	3 (3.1)	2 (3.8)	5 (3.3)	58 (62.4)	20 (41.3)	78 (55.7)
Unemployed	0 (0)	0 (0.0)	0 (0.0)	1 (1.1)	2 (4.3)	3 (2.1)
Invalid pensioner	48 (49.0)	21 (39.6)	69 (45.7)	2 (2.2)	3 (6.5)	5 (3.6)
Old-age pensioner	47 (48.0)	30 (56.6)	77 (51.0)	3 (3.2)	18 (39.1)	21 (15.0)
Other	0 (0)	0 (0.0)	0 (0.0)	29 (31.1)	4 (6.5)	33 (23.6)
**Children** N (%)						
Yes	84 (85.7)	48 (90.6)	132 (87.4)	74 (79.6)	41 (87.2)	115 (82.1)
**Length of diseases/care**						
Mean (SD)	16.4 (11.6)	10.1 (7.7)	14.1 (10.8)	10.2 (8.8)	7.33 (8.5)	9.21 (8.7)
Min-max	2–37 years	2–30 years	2–37 years	1–25 years	0–22 years	0–25 years
**Disease**						
Multiple sclerosis	54 (55.1)	34 (64.2)	88 (58.3)	51 (54.8)	31 (65.9)	82 (58.7)
Parkinson’s	42 (42.9)	16 (30.2)	58 (38.4)	40 (43.0)	13 (27.7)	53 (37.6)
Motor neuron	2 (2.0)	3 (5.6)	5 (3.3)	2 (2.2)	3 (6.4)	5 (3.7)

### Evaluation of the difference in quality of life between the intervention and control groups

First, we assessed the difference between the intervention and control groups during the first and second measurements (see Table 2). Before the intervention (Time I), no statistically significant differences were found in the evaluation of the individual domains of the quality of life of the patients with PND and family caregivers included in the intervention and control samples. In the second measurement (Time II), a statistically significant difference in certain parameters was found in the sample of patients. Following intervention, patients from the intervention group had a lower symptoms burden (*p* < 0.001), and a more positive evaluation of emotional functioning (*p* < 0.001), social functioning (*p* = 0.046), spiritual area (nonreligious) (*p* = 0.050), and global QoL (*p* = 0.023). Similarly, the family caregivers from the intervention group had a lower symptoms burden (p < 0.001), better emotional functioning (*p* = 0.016), and spiritual area (nonreligious) (*p* = 0.042), and a more positive evaluation of global QoL (*p* = 0.001) and global health (*p* = 0.002).

**Table 2:** Comparison of differences regarding evaluation of individual quality of life domains of patients and family caregivers in intervention and control samples during first and second measurements

	Time I				Time II			
	Interven. N = 98	Control N = 53	Diff. x̄	p*	Interven. N = 98	Control N = 53	Diff. x̄	p*
**Patients with PND**	x̄ (s)	x̄ (s)			x̄ (s)	x̄ (s)		
Symptoms burden	41.2 (16.2)	43.4 (16.2)	2.2	0.287	35.8 (15.7)	46.3 (18.3)	10.5	**< 0.001**
Activities of daily living	52.6 (21.9)	52.2 (21.9)	−0.4	0.925	50.0 (19.9)	51.7 (23.1)	1.7	0.539
Emotional functioning	40.9 (19.4)	43.3 (16.7)	2.4	0.282	33.9 (19.2)	43.9 (17.7)	10.0	**< 0.001**
Social functioning	47.1 (17.1)	47.8 (16.8)	0.7	0.874	43.5 (14.7)	50.1 (17.3)	6.6	**0.046**
Spiritual (nonreligious)	50.2 (18.1)	51.8 (19.0)	1.6	0.873	46.6 (19.1)	53.7 (19.2)	7.1	**0.050**
Spiritual (religious)	42.2 (15.6)	43.2 (13.9)	1.0	0.905	42.8 (14.6)	45.3 (15.1)	2.5	0.264
Global QoL	4.5 (1.6)	4.4 (1.6)	−0.1	0.834	5.3 (1.7)	4.6 (1.9)	− 0.7	**0.023**
Global health	4.2 (1.5)	4.5 (1.7)	0.3	0.351	4.9 (1.5)	4.7 (1.8)	−0.2	0.410
**Family caregivers** N = 93		N = 47			N = 93	N = 47		
Symptoms burden	8.8 (7.7)	9.4 (5.7)	0.6	0.129	6.1 (6.8)	9.4 (6.4)	3.3	**< 0.001**
Activities of daily living	34.5 (25.6)	27.1 (24.3)	−7.4	0.106	25.1 (22.4)	23.2 (21.6)	−1.9	0.616
Emotional functioning	31.6 (24.3)	33.6 (18.3)	2.0	0.294	25.5 (21.8)	33.7 (20.2)	8.2	**0.016**
Social functioning	45.6 (19.6)	40.5 (14.7)	−5.1	0.061	40.8 (17.7)	41.7 (18.5)	0.9	0.696
Spiritual (nonreligious)	30.6 (20.7)	30.8 (17.6)	0.2	0.806	26.2 (20.4)	32.4 (17.5)	6.2	**0.042**
Spiritual (religious)	38.7 (19.1)	40.7 (15.5)	2.0	0.996	40.0 (18.1)	40.4 (17.0)	0.4	0.969
Global QoL	6.7 (2.3)	6.2 (2.2)	−0.5	0.107	7.3 (2.0)	6.3 (1.9)	−1.0	**0.001**
Global health	6.9 (2.3)	6.2 (2.2)	−0.7	0.063	7.4 (2.1)	6.3 (1.9)	−1.1	**0.002**

*Mann-Whitney U test

### Evaluation of the difference in quality of life between first and second measurements

Differences between the first and second measurements were assessed in the intervention and control groups (see Table 3). The patients and the family caregivers from the intervention group scored lower (had better quality of life) on the symptomatic scale (*p* < 0.001) and in all domains of the functional scale except the spiritual area/religious domain. After intervention (Time II) patients and family caregivers evaluated global quality of life (p < 0.001) and global health (p < 0.001) more positively. On the other hand, the patients and family caregivers from the control sample did not demonstrate any statistically significant changes.

**Table 3:** Comparison of differences regarding evaluation of QoL domains by patients and family caregivers in intervention and control samples during first and second measurements

PNQOL	Interventional				Control			
	Time I x̄ (s)	Time II x̄ (s)	Diff, x̄	p*	Time I x̄ (s)	Time II x̄ (s)	Diff. x̄	P*
**PATIENTS WITH PND**								
Symptoms burden	41.2 (16.2)	35.8 (15.7)	−5.4	**< 0.001**	43.4 (16.2)	46.3 (18.3)	2.9	0.125
Activities of daily living	52.6 (21.9)	50.0 (19.9)	−2.6	**0.021**	52.2 (21.9)	51.7 (23.1)	−0.6	0.837
Emotional functioning	40.9 (19.4)	33.9 (19.2)	−7.0	**< 0.001**	43.3 (16.7)	43.9 (17.7)	0.6	0.747
Social functioning	47.1 (17.1)	43.5 (14.7)	−3.6	**< 0.001**	47.8 (16.8)	50.1 (17.3)	2.3	0.159
Spiritual (nonreligious)	50.2 (18.1)	46.6 (19.1)	−3.6	**0.001**	51.8 (19.0)	53.7 (19.2)	1.9	0.178
Spiritual (religious)	42.2 (15.6)	42.8 (14.6)	0.6	0.260	43.2 (13.9)	45.3 (15.1)	2.1	0.112
Global QoL	4.5 (1.6)	5.3 (1.7)	0.8	**< 0.001**	4.4 (1.6)	4.6 (1.9)	0.2	0.457
Global health	4.2 (1.5)	4.9 (1.5)	0.7	**< 0.001**	4.5 (1.7)	4.7 (1.8)	0.2	0.303
**FAMILY CAREGIVERS**								
Symptoms burden	8.8 (7.7)	6.1 (6.8)	−2.7	**< 0.001**	9.4 (5.7)	9.4 (6.4)	0.0	0.735
Activities of daily living	34.5 (25.6)	25.1 (22.4)	−9.4	**< 0.001**	27.1 (24.3)	23.2 (21.6)	−3.9	0.691
Emotional functioning	31.6 (24.3)	25.5 (21.8)	−6.1	**< 0.001**	33.6 (18.3)	33.7 (20.2)	0.1	0.693
Social functioning	45.6 (19.6)	40.8 (17.7)	−4.8	**< 0.001**	40.5 (14.7)	41.7 (18.5)	1.2	0.348
Spiritual (nonreligious)	30.6 (20.7)	26.2 (20.4)	−4.4	**0.003**	30.8 (17.6)	32.4 (17.5)	1.6	0.421
Spiritual (religious)	38.7 (19.1)	40.0 (18.1)	1.3	0.107	40.7 (15.5)	40.4 (17.0)	−0.3	0.739
Global QoL	6.7 (2.3)	7.3 (2.0)	0.6	**< 0.001**	6.2 (2.2)	6.3 (1.9)	0.1	0.661
Global health	6.9 (2.3)	7.4 (2.1)	0.5	**< 0.001**	6.2 (2.2)	6.3 (1.9)	0.1	0.699

*Wilcoxon singed-rank test

### Evaluation of predictors of change in quality of life

The results of the linear regression analysis can be found in Table 4. Only domains in which the model was statistically significant are depicted. In all QoL domains, the predictor of change was confirmed to be the provided intervention. Furthermore, in certain domains, gender and marital status (patients’ sample), and marital status and patients’ PPS score (family caregivers sample) were additional predictors of change.

**Table 4:** Predictors of change in individual quality of life domains

Independent variables	Dependent variables									
	Total QoL		Total health		D2		D3		D4a	
**PATIENTS**	**ß**	**p**	**ß**	**p**	**ß**	**p**	**ß**	**p**	**ß**	**p**
Intervention	−0.194	**0.025**	−0.199	**0.020**	0.273	**0.002**	0.308	**0.000**	−0.276	**0.001**
Gender	0.212	**0.016**	0.226	**0.009**	−.0068	0.430	−0.118	0.169	0.132	0.120
Age	−0.075	0.386	−0.060	0.485	0.007	0.932	−0.152	0.078	−0.065	0.441
Marital status	0.111	0.620	0.193	**0.024**	−0.197	**0.022**	−0.133	0.118	0.236	**0.006**
PPS	0.043	0.198	0.037	0.663	0.018	0.836	−0.023	0.783	−0.005	0.949
Model sum.		**0.026**		**0.007**		**0.014**		**0.004**		**0.002**
**FAMILY CAREGIVERS**										
Intervention	0.254	**0.048**	0.282	**0.041**	−0.249	**0.002**	−0.268	**0.002**	−0.188	**0.033**
Gender	0.013	0.876	0.025	0.777	0.113	0.159	0.013	0.874	0.024	0.784
Age	0.116	0.213	0.151	0.122	−0.171	0.057	−0.108	0.246	−0.094	0.330
Marital status	−0.267	**0.005**	−0.250	**0.011**	0.344	**0.000**	0.279	**0.003**	0.158	0.103
Patients PPS	0.269	**0.002**	0.075	0.389	−0.213	**0.008**	− 0.156	0.062	− 0.177	**0.043**
Model sum.		**0.001**		**0.048**		**0.000**		**0.001**		**0.040**

D2 – emotional functioning, D3 – social functioning, D4a – spiritual area (nonreligious)

### Evaluation of differences regarding satisfaction with provided care

When assessing their satisfaction with the provided care, patients with PND and family caregivers from the intervention group reported significantly higher satisfaction with the provided care in all evaluated areas (see Table 5).

**Table 5:** Comparison of satisfaction-with-care evaluation of intervention and control groups

	Patients with PND				Family caregivers			
Satisfaction with care	Intervention x̄ (s)	Control x̄ (s)	Difference x̄	p*	Intervention x̄ (s)	Control x̄ (s)	Difference x̄	P*
1_ Overall satisfaction	86.8 (14.8)	80.0 (15.8)	−6.8	**0.009**	88.5 (14.6)	80.5 (20.2)	−8.0	**0.025**
2_Relation to doctor	87.3 (13.2)	79.8 (14.6)	−7.5	**0.002**	86.5 (15.2)	78.8 (20.7)	−7.7	**0.040**
3_Health worker care	85.9 (13.6)	79.7 (14.9)	−6.2	**0.020**	88.1 (13.1)	81.3 (18.3)	−6.8	**0.053**
4_Disease management	59.7 (23.9)	52.8 (20.9)	−6.9	**0.045**	87.7 (12.8)	79.5 (17.0)	−8.2	**0.009**
5_Communication/de-cisions	85.6 (14.5)	77.9 (14.7)	−7.7	**0.002**	87.3 (13.3)	79.5 (14.2)	−7.8	**0.002**

## Discussion

Palliative care in neurology has been a focus of discussion in recent years [4–9]. Applying general and specialized palliative care to patients with PND may benefit both the patients [14–18] and their family caregivers [16]. Specialized palliative care offers the possibility of consultations with a palliative team. In our study, we analyzed the benefits of consultations with a multidisciplinary team in connection with QoL and satisfaction with care in patients with PND and their family caregivers. Multidisciplinary team consultations were provided in the care facilities of the patients with PND. Evaluating the efficacy of palliative services and programs is one of the priorities of current international research regarding palliative care [24] of patients with PND and other diseases. There are two major reasons for the assessment of quality of life in research and clinical practice within palliative care. First, quality of life is an input variable of the level of impact of certain diseases and their treatment on the life of patients, and, second, it is an output variable representing the level of success of certain interventions [34]. In our study, we viewed the evaluation of quality of life as an output variable; thus reflecting the level of success of the provided intervention – i.e., consultations with the multidisciplinary palliative care team. In our sample, patients in advanced stages of disease were included (Palliative Performance Scale ≤70), i.e., when their disease had significantly limited their mobility, ability to work or activities at home, and substantial symptoms had manifested themselves. Although patients were not in terminal stages of disease, they could be provided with early palliative care, as recommended for patients with PND [6, 35], to improve symptom management and the well-being of patients and their families [16, 19].

### Main findings

The most significant finding of our research is the confirmation of the positive effect of the intervention (i.e., consultations with the neuropalliative multidisciplinary team) provided to patients with PND and their family caregivers on quality of life in almost all areas. Consultations with a multidisciplinary palliative team may help patients with PND to change their subjective view of quality of life in a positive way. Mitigating the symptoms burden, changing the importance of quality of life domains, and changing the perception of the control patients have over certain areas of their quality of life, or, alternatively, learning to accept and live with reduced quality of life, may help patients evaluate subjective quality of life more positively [36]. These topics may be discussed by patients and family caregivers, within the psychosocially supportive environment provided by the multidisciplinary team.

On the other hand, no association was found between the provided intervention and spiritual area (religious). Neither patients with PND nor family caregivers wished to discuss this area during consultations with the multidisciplinary team. An improvement was identified in the non-religious area, which included evaluation of the degree of meaningfulness of life, composure, control over one’s own life, and desperation.^31^ This may be due to the fact that the Czech Republic is largely an atheist country [36], in which non-religious topics may be more commonly discussed as part of spiritual care. A connection between palliative care and spirituality has been confirmed in a number of studies [37].

We also studied the predictors of change in quality of life. The provided intervention was confirmed as a predictor, and in some domains, gender (global health, global QoL) and marital status (emotional functioning, global health, spiritual area (nonreligious)) were also predictors. While the functional state of the patients, determined through the PPS scale, was not confirmed as a predictor for patients, it was confirmed as such for family caregivers. A worse functional state in patients represented a greater burden for family caregivers.

Another important finding of our research is the fact that neuropalliative care improves satisfaction with provided care for both patients with PND and their family caregivers. A significant difference in evaluation was determined in the domain “**disease management**”, in which patients and the family caregivers were asked questions regarding their satisfaction with the treatment, resolution of physical and emotional problems, and succession of care, and services provided in the household of the patient. A multidisciplinary approach may help patients resolve not only the physical problems resulting from the disease but also any emotional issues, and it may help secure successive health/social care based on the individual needs of the client [1]. However, on the basis of a literature overview, Aspiral et al. [25] warn that using satisfaction as a method of evaluating the quality of health services can be problematic. One of the reasons is that satisfaction is under-theorized and no widely accepted definition exists. However, satisfaction with care can be important for the quality of life of patients and their family caregivers. That is the reason why is appropriate to measure it.

An important recommendation of our research is that neuropalliative care should be used for patients with PND before they reach the terminal stage of the disease.

### Limitations

A limitation of the study was the uneven number of respondents in the intervention and control groups, caused by random categorization of patients according to the last digit of their social security number. This was intended to prevent patients more likely to improve after intervention from deliberately being included in the intervention group.

Other limitations of the study were the low number of patients with MND, due to the unwillingness of MND patients to participate in the research and inclusion criterion for patients PPS ≤70. Our study aimed to analyze the effect of neuropalliative care of patients with PND in the advanced stage of the disease. The patients with PPS = 70 points are questionable limits for the advanced stage of disease. Reduced ambulation is located at the PPS 70% and PPS 60% level. This reduction of ambulation is tied to the patient’s inability to carry out their normal job, work occupation, or some hobbies or housework activities. The patients with PPS 70% is still able to walk and transfer on their own. Intake is normal or reduced. We included these patients (PPS = 70) because they may have burden symptoms and may need palliative care support. The second reason is that often symptom of many PND is mental decline and the patients need palliative consultation early. Despite the stated limitations, the study produced useful results, confirming the efficiency of neuropalliative care.

## Conclusion

Providing palliative care to patients with PND in the form of consultations with a multidisciplinary palliative team may help patients and their family caregivers maintain, and possibly improve, evaluation of quality of life of patients and their family caregivers. For further research, we recommend analysis of the benefits of different forms of general and palliative care for patients with PND on a larger sample of respondents, and that a long-term study be conducted to determine the connection between palliative intervention and survival. We believe it would be worthwhile to educate doctors (particularly neurologists) and, possibly, other healthcare workers, about the possible utility of palliative care in neurology.

## Data Availability

The datasets used and/or analyzed during the current study are available from the appropriate author on reasonable request.

## Abbreviations

ADL: Activities of Daily Living
EDSS: Expanded Disability Status scale QoL – quality of life
EFNS: European Federation of Neurological Societies
MND: Motor neuron disease
MS: Multiple sclerosis
PD: Parkinson’s disease
PNDQoL: Progressive Neurological Diseases Quality of Life
PND: Progressive neurological disease
PPS: Palliative Performance Scale
